# Our Environment Shapes Us: The Importance of Environment and Sex Differences in Regulation of Autoantibody Production

**DOI:** 10.3389/fimmu.2018.00478

**Published:** 2018-03-08

**Authors:** Michael Edwards, Rujuan Dai, S. Ansar Ahmed

**Affiliations:** ^1^Department of Biomedical Sciences and Pathobiology, Virginia-Maryland College of Veterinary Medicine, Virginia Tech, Blacksburg, VA, United States

**Keywords:** sex hormones, lupus, microbiota, endocrine disrupting chemicals, DNA methylation, epigenetics

## Abstract

Consequential differences exist between the male and female immune systems’ ability to respond to pathogens, environmental insults or self-antigens, and subsequent effects on immunoregulation. In general, females when compared with their male counterparts, respond to pathogenic stimuli and vaccines more robustly, with heightened production of antibodies, pro-inflammatory cytokines, and chemokines. While the precise reasons for sex differences in immune response to different stimuli are not yet well understood, females are more resistant to infectious diseases and much more likely to develop autoimmune diseases. Intrinsic (i.e., *sex hormones, sex chromosomes, etc*.) and extrinsic (*microbiome composition, external triggers, and immune modulators*) factors appear to impact the overall outcome of immune responses between sexes. Evidence suggests that interactions between environmental contaminants [e.g., endocrine disrupting chemicals (EDCs)] and host leukocytes affect the ability of the immune system to mount a response to exogenous and endogenous insults, and/or return to normal activity following clearance of the threat. Inherently, males and females have differential immune response to external triggers. In this review, we describe how environmental chemicals, including EDCs, may have sex differential influence on the outcome of immune responses through alterations in epigenetic status (such as modulation of microRNA expression, gene methylation, or histone modification status), direct and indirect activation of the estrogen receptors to drive hormonal effects, and differential modulation of microbial sensing and composition of host microbiota. Taken together, an intriguing question develops as to how an individual’s environment directly and indirectly contributes to an altered immune response, dysregulation of autoantibody production, and influence autoimmune disease development. Few studies exist utilizing well-controlled cohorts of both sexes to explore the sex differences in response to EDC exposure and the effects on autoimmune disease development. Translational studies incorporating multiple environmental factors in animal models of autoimmune disease are necessary to determine the interrelationships that occur between potential etiopathological factors. The presence or absence of autoantibodies is not a reliable predictor of disease. Therefore, future studies should incorporate all the susceptibility/influencing factors, coupled with individual genomics, epigenomics, and proteomics, to develop a model that better predicts, diagnoses, and treats autoimmune diseases in a personalized-medicine fashion.

## Introduction

The incidence of autoimmune and allergic diseases has been increasing for multiple decades (1, 2). Despite intensive studies in many laboratories, the etiology of autoimmune diseases is not well understood. It is nevertheless clear that there is no single genetic factor that solely determines the susceptibility to autoimmune diseases. Rather, susceptibility to autoimmune diseases appears to involve complex interactions of genetic, epigenetic, hormonal, and environmental factors. Many (but not all) autoimmune diseases preferentially demonstrate a female dominant susceptibility bias. The high female to male incidence ratios in autoimmune diseases such as autoimmune thyroiditis, systemic lupus erythematosus (SLE), and Sjögren’s syndrome in both humans and relevant animal models have been widely reported (3–8). Interestingly, even those diseases that did not show a strong female bias of susceptibility in the past, such as multiple sclerosis (MS), now appear to tilt toward female predisposition. Patients diagnosed with MS were initially reported to have close to a 1:1 female:male ratio in the 1950s (9). This ratio increased to 2:1 in the 1980s (10), and further to 3:1 in recent reports (11). While the precise reasons for sex differences are not known, the potential contribution of changes in environmental factors remains an intriguing possibility. The implication of non-genetic factors (e.g., epigenetic and environmental factors) is also evident in studies that reported the concordance rate of monozygotic twins manifesting autoimmune diseases is only between 20 and 35% (12–15). Further evidence for an environmental component driving autoimmune pathology exists with the Gullah population in South Carolina who are genetically very similar to members of their ancestral home of Sierra Leone. In a recent report, while the SLE disease prevalence (as measured by serum antinuclear antibodies) in the Gullah population is similar to their African counterparts, notably, the African cohort had higher levels of circulating anti-Smith and anti-cardiolipin autoantibodies, as well as increased numbers of seropositive individuals to multiple viral infections (16). This suggests that in genetically very similar populations, environmental factors can promote autoantibody production. The potential contribution of differences in exposure to environmental chemicals between these two population groups cannot be discounted. Interestingly, human SLE patients with pet dogs are more likely to have dogs that also suffer from SLE (17). This finding supports the claim that a transmissible or common environmental agent, or agents, may be present that increased the risk for SLE development within the human and canine populations. Even in genetically susceptible inbred mice that spontaneously develop autoimmune diseases, such as lupus, differences in the outcome or severity of the diseases has been noted among various laboratories (18–24). This supports non-genetic environmental factors influence on autoimmune disease.

It is now recognized that sex differences in the immune system cannot be solely attributable to differences in sex chromosomes and sex hormones (6, 7, 25). Direct comparisons among various studies exploring the specific mechanisms underlying the observed female bias in many autoimmune disorders are difficult due to differences in study methodology, population cohorts, and various extrinsic factors unable to be controlled for in human populations. Nevertheless, when the data are explored as a whole, the consequence of these variations can be mitigated and trends can be identified regarding sex-based differences in multiple systems.

**Figure 1:**
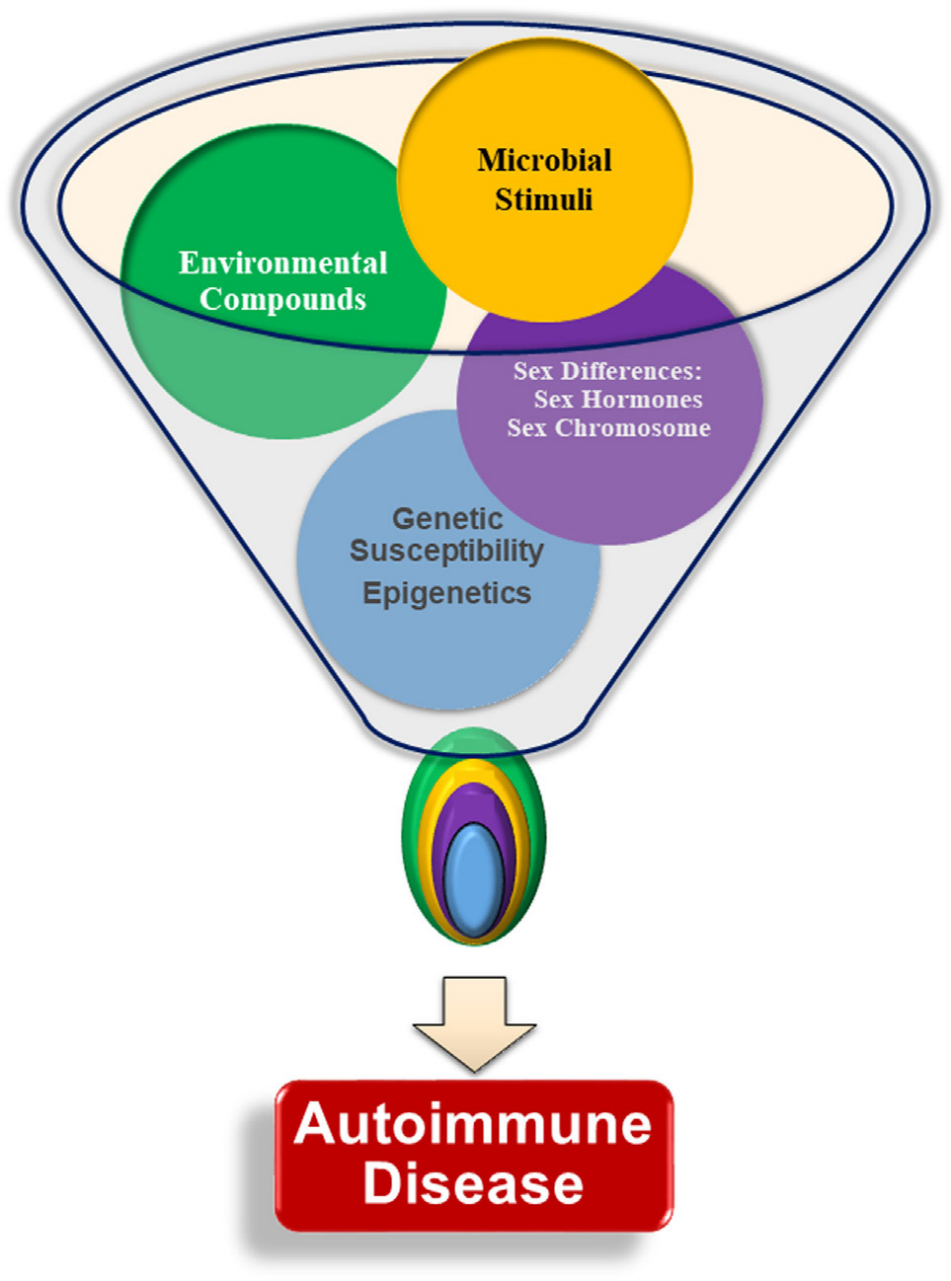
Interactions of multiple factors are required for the development of autoimmunity. Genetic susceptibility, sex chromosomes, sex hormones, infections and microbial stimuli, and environmental factors are all thought to contribute to autoimmune pathogenesis and lead to autoantibody production. There is little evidence that any one particular factor is able to initiate autoimmunity without input from another factor. The specific relationships and interplay among each of the various factors, such as the relative importance of one factor compared with the others, age at, or duration of, exposure, is not yet understood.

In general, normal healthy males are thought to have immune systems that maintain tolerance, while the female immune system is susceptible to break in immune tolerance as evidenced by higher production of autoantibodies (26–28). Sex chromosomes contribute genetic differences, with multiple genes involved in immune system responses present on the X-chromosome, including genes for FoxP3 and toll-like receptors (TLRs) 7 and 8. These genes can be differentially expressed in males and females due to incomplete X-chromosome inactivation in the females, leading to potentially increased gene expression in females (29). Steroidal sex hormone levels vary between sexes, with female predominant estrogens promoting B cell survival and contributing to exacerbation of multiple autoimmune diseases, and androgens exerting immune regulatory effects to prevent, suppress, or delay autoimmunity (30, 31). Endocrine disrupting chemicals (EDCs) act through multiple mechanisms, displaying both estrogenic and anti-estrogenic properties, reducing androgen production, and influencing epigenetic regulation (28, 32). It is now prudent to incorporate environmental influences (e.g., EDCs) in studying the development of autoimmune diseases, which will provide a more comprehensive understanding of mechanisms of autoimmune diseases (33). Assessed individually, these various factors may not be able to induce autoimmunity sufficiently. However, when multiple internal and environmental factors interact, these may cause the loss of tolerance, the production of autoantibodies, and drive autoimmune disease pathogenesis (Figure 1). This review will focus on how sex differences identified in genetics, epigenetics, hormonal responses, and response to microbial stimuli influence immune tolerance dysregulation and autoantibody production, with an emphasis on the contributing effects of EDCs on immunological functions.

## Sex Differences in Genetics and Autoimmunity

Female cells are genetically the same as male cells in all chromosomes except the sex-specific X and Y chromosomes. To compensate for gene copy differences, female cells, other than egg cells, undergo X-chromosome inactivation, thereby permanently silencing one copy of the X chromosome. This process may be incomplete in some individuals, leading to overexpression of genes present on the X-chromosome. Abnormalities in chromosome numbers may exist, such as in Klinefelter syndrome, where males have one or more extra X chromosomes. Notably, men with Klinefelter syndrome are predicted to have a similar risk of SLE to that of females, and a 14-fold increase in SLE risk compared with healthy males (34). It is conceivable that in the context of incomplete X-chromosome inactivation, females could have alterations in the expression of X-chromosome linked genes that promote inflammation and subsequent autoimmunity, such as TLR7/8.

Many autosomal genes are differentially expressed in males and females. The transcription factor vestigial-like family 3 (VGLL3) was recently found to be upregulated in female tissues, such as ovaries, the uterus, adipose tissue, and smooth muscle. *VGLL3* is located on chromosome 3, and it is unknown at this time what contributes to this sexual differential expression pattern. This transcription factor contributes to the differential expression of hundreds of genes between sexes. Genes of interest regulated by VGLL3 include *BAFF, ITGAM, IL-7, ICAM-1, MMP9*, and *ETS1*. These female biased genes are associated with known autoimmunity susceptibility loci and inflammatory processes, and the increased expression of these genes appears independent of sex hormone regulation (35). Furthermore, it is also possible that other newly identified and unknown transcription factors are contributing to the sex bias gene expression and autoimmune disease susceptibility.

## Sex Hormones and Environmental EDC Regulation of Immunity and Autoimmunity

**Figure 2:**
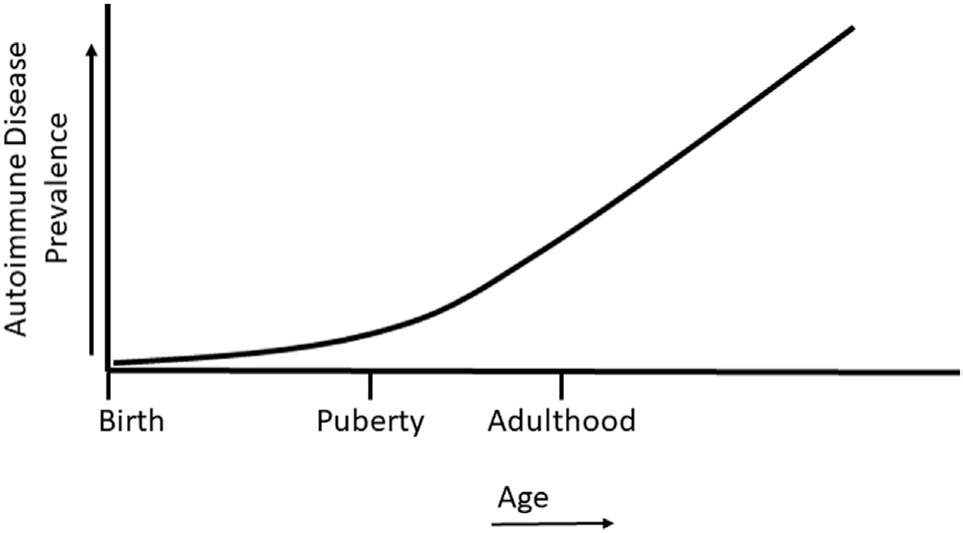
Autoimmune disease prevalence in relation to life stage. Autoimmune diseases can develop during childhood, but most autoimmune diseases develop following the onset of puberty and in later life.

Sex differences in sex steroid hormone levels and regulation on the immune system of normal and autoimmune individuals have been extensively studied (7, 36–40). While the sex differential effects on immunity and autoimmunity cannot be solely attributable to sex hormone profiles, sex steroid hormones do have a major impact on various aspects of the immune system, including their contribution to cell differentiation, cytokine profiles, epigenetic alterations, and autoimmune disease (36, 38–44). The case for the role of sex hormones in autoimmune diseases can be further made by the fact that a majority of autoimmune diseases are manifested after sexual maturity, at a time when sex hormone levels are elevated and differential biological responses of sex-hormone regulated genes are evident (Figure 2). Interestingly, it is not yet understood why women are at a higher risk of developing autoimmune diseases such as SLE, rheumatoid arthritis, Graves’ disease, and thyroiditis following menopause (45). EDCs are able to exert agonistic or antagonistic roles on normal physiological sex hormone actions, enhancing or mitigating hormonal effects on immune cells (25, 46–48). As with many endocrine components, sex hormones and EDCs exert differential effects, not only due to dosage but also in temporally dependent and context-specific manners.

Exposure to EDCs is nearly impossible to avoid in current societies. These compounds can be present in drinking water, cosmetic products, paper products, food and beverage containers, many forms of plastics, and the food we eat (32). The route and dosage of exposure are important considerations when determining the effect EDCs will have on various aspects of health and physiology. Many EDCs have been determined to be able to elicit bi-phasic dose responses, with evidence that very low EDC concentrations can exert a positive effect, while at higher concentrations they may have opposite effects, and *vice versa*. Currently, controversy exists regarding evaluation of internal concentrations, metabolites, and daily exposure levels of EDCs (49).

Little consensus has been reached regarding when, where, and how EDCs disrupt endocrine homeostasis in exposed individuals. One vital issue that impairs our understanding of the mechanisms and overall influence of EDCs on health is the potential lag between exposure and development of clinical signs, such as reproductive disorders. In humans, the lag period may be years or decades before sexual maturity and fertility can be tested (50). Much of the current data on EDC functions and effects are targeting alterations in reproductive systems. Due to the wide variety of compounds and exposure routes, this review will only address how well-studied models of EDCs, such as bisphenol-A (BPA) and phytoestrogens, may affect the immune system.

### Estrogen, Natural, and Environmental

The effects of estrogen on immune cell populations and functions have been extensively studied and reviewed (6, 36–40). We will highlight the important aspects of estrogen’s actions that promote or inhibit autoantibody production. Estrogens are able to exert effects on multiple immune cell phenotypes through activating either estrogen receptors (ERs)-mediated genomic signaling or G protein-coupled estrogen receptor 1 (GPR30/GPER1)-coupled non-genomic signaling pathways (30). Following ligand binding, ERα and/or ERβ binds to the estrogen response element (ERE), which drives transcriptional regulation, particularly Pax5, BSAP, HOXC4/HoxC4, and activation-induced cytidine deaminase (AID) genes in B cells, promoting B cell maturation and survival. Estrogen activated GPR30 signals through P38/ERK MAPK and PI3 kinase pathways, driving B cell activation and rearrangement of the Ig heavy and light chain, as well as activating NF-κB (30). Furthermore, sex hormones and hormone metabolites can also induce their effects on target cells (such as cells of the immune system) in sex-hormone receptor independent mechanisms (51, 52). Activated ERs can bind to other transcription factors (such as NF-κB) to mediate cell signaling for regulating gene expression. In addition, ERs can be activated independent of ligand binding (53, 54). Therefore, it is conceivable that, in females, direct and indirect activation of ERs by external triggers, such as endocrine disruptors, can potentially have differential effects compared with males.

**Figure 3:**
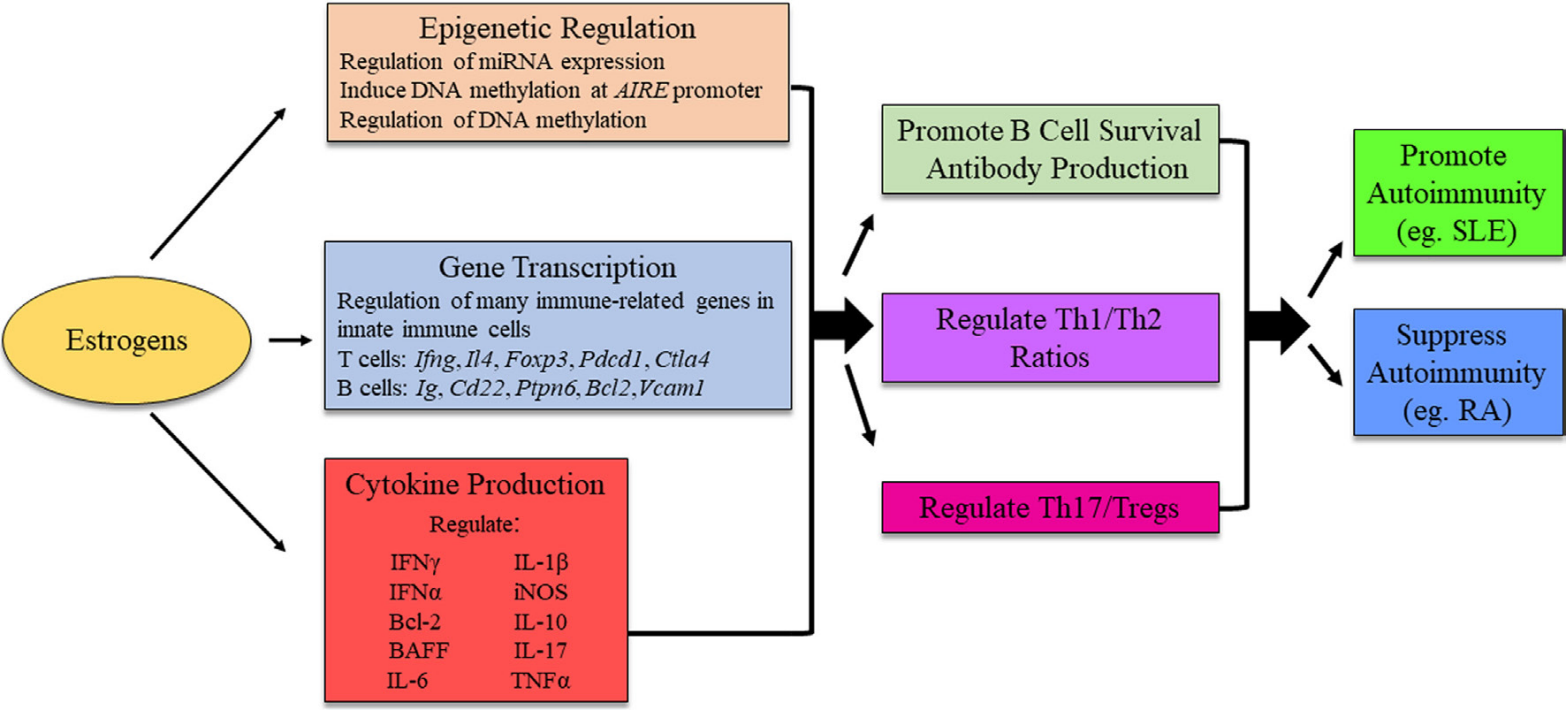
Possible mechanism for estrogens to influence immunity and autoimmune disease development. The exact mechanism for estrogenic influence on autoimmune disease development is likely disease- and context-dependent, and research is ongoing to identify distinct pathways in which estrogen is able to exert its effects. The figure illustrates potential molecular mechanisms of estrogen regulation. *AIRE*, autoimmune regulator.

In most instances, estrogen enhances both cell-mediated and humoral immunity. Studies in peroxisome proliferator-activated receptor (PPAR) knockout mice show that T follicular helper cell responses, important for antibody production, were upregulated in female but not in male CD4-PPARγ^KO^ mice, in part due to estrogen (55). In regards to B cells and antibody production, estrogen drives B cell maturation, immunoglobulin class switch recombination, and somatic hypermutation in germinal centers, promotes B cell survival, and enhances antibody production (30, 56, 57). Directly, estrogen regulates gene transcription through ERs binding to ERE sites. Indirectly, estrogen promotes B cell survival through increased B-cell-activating factor (BAFF) production. In mouse models, BAFF gene expression is upregulated by estrogens and interferon (IFN) stimulation both at the mRNA and protein levels through a mechanism involving ERα, IRF5, or STAT1. Treatment of the mouse macrophage cell line RAW264.7 with IFNα, IFNγ, or estrogen induced p202, which correlated with increased BAFF production, contributing to sex differences (58). In humans, under normal physiological conditions, no detectable differences are found between male and female BAFF levels. However, following estradiol treatment, both sexes had an increase in BAFF production, with the increase in females being much more profound (59). Thus, in the presence of estrogen, B cell survival, and maturation is enhanced through multiple mechanisms, potentially increasing the ability of autoreactive B cells to break tolerance and drive autoantibody production (Figure 3).

BALB/c transgenic mice treated with estrogen had increased Bcl-2 production that allowed naïve B cells to break tolerance induction and drive anti-dsDNA autoantibody production (60). Anti-cardiolipin autoantibody was shown to be enhanced in orchiectomized male and normal female B6 mice treated with 17β-estradiol, but replacement of dihydrotestosterone (DHT) in castrated males had no effect, and intact males had lower levels of circulating autoantibodies than females (43). Autoreactive B cell pools are created predominantly in females, and ER signal-mediated activation of DCs was found to modulate T and B cell responses (61–63). Enhanced B cell survival through estrogen’s various actions promotes self-reactive B cell escape from negative selection in the bone marrow, and progression of autoantibody production (64, 65). Therefore, it is conceivable that the lower levels of circulating E2 in males, in combination with higher levels of androgens, allows for better regulation and removal of autoreactive B cell populations before autoimmunity onset.

Evolutionarily, the female immune system is biologically equipped to robustly respond to infectious threats to protect the young dependent offspring and in the larger sense aiding in the survival of species. With the passage of time in the relatively recent era, introduction of new chemicals and emerging and re-emerging infections now pose unique challenges to the primed female immune system. It can be argued that initially the female immune system had biologically been exposed to natural estrogens. With the advent of, and exposure to, EDCs, the female immune system may be exposed to “surges or overloads” of endocrine compounds with competing endocrine effects, thus increasing the chance for deviation of immune modulation by hormones. Whether these new threats have contributed to increased autoimmune diseases is an open question that warrants investigation.

The ability of EDCs to influence the immune system subsets and alter disease susceptibility is poorly understood at this time. BPA has multiple estrogenic-like functions that alter T cell subset, B cell function, and dendritic cell activity, inducing abnormal immune signaling and disrupting ER and PPAR signaling, thus, altering target gene transcription. In mice, BPA induced splenocyte proliferation and shifted the cytokine profile from Th2- to Th1-mediated cytokines, enhancing autoimmunity (32). For example, BPA has been associated with the development of type 1 diabetes mellitus (T1DM) in non-obese diabetic (NOD) mice, a mouse model of insulitis and leukocytic infiltration of pancreatic islets leading to type 1 diabetes mellitus (66–68). Human studies have also associated EDCs with the development of organ-specific autoimmune diseases mediated by autoreactive T cells. For example, serum BPA levels correlated with increased antithyroperoxidase in human patients of Hashimoto’s thyroiditis, and estrogens regulate miR-21, which may drive inflammation in polymyositis (69, 70). Exaggerated T cell activation and polar Th1/Th2 shifts are due in part to increased antigen-specific IFNγ following BPA exposure (71). In this way, BPA shows an antagonistic effect to physiological estrogen responses. BPA can affect the MAPK and STAT pathways, disrupting the normal prevention of autoreactive T cell proliferation and survival (72). IFN-associated mechanisms modulated by BPA have been shown to influence SLE pathogenesis (73). Interestingly, prenatally BPA exposed mice showed an increase in IL-4 and IFNγ. However, mice exposed after reaching adulthood showed increases in IL-4, IL-10, and IL-13, but not IFNγ. In both cases, Tregs were reduced (74). This disparity shows that the effects of EDCs are strongly dependent on age at exposure.

Bisphenol-A has been shown repeatedly to increase immunoglobulin production in B cells. B1 cells have been associated with Sjögren’s syndrome and rheumatoid arthritis patients (75–77), and in mouse models of SLE have been shown to be more sensitive to EDC’s modulatory effects than B2 cells (78). B1 cells increased production of anti-dsDNA autoantibody, enhanced IgG deposition and glomerulonephritis and overall worsened SLE signs following BPA implantation (79). MRL/*lpr* mice fed diets that contained phytoestrogen compounds diadzin and genistin had higher levels of IgG and complement component C3 deposition in glomeruli, along with altered immune cell infiltration into glomeruli compared with mice fed a diet devoid of estrogenic components (23). The majority of evidence supports that exposure to EDCs enhances autoantibody production and autoimmunity in mouse models of disease.

The complex immunological effects of EDCs can also display immune-suppressive effects. In people under 18 years of age, circulating BPA levels were negatively associated with anti-cytomegalovirus (CMV) antibody titers, suggesting that some EDCs may attenuate antiviral immunity (80). Short-term BPA exposure in New Zealand Black/White F1 progeny (NZB/W_F1_) mice suppressed autoimmunity, reduced albuminuria, and exten-ded the disease-free period, through modulation of IFNγ (81). Thus, when determining the impact of EDC exposure on disease states, it is vital to view data in the context of dosage, exposure length, age, and infection status, due to the wide range of effects that may be altered by EDC exposure.

To date, less is known regarding the role EDC exposure has on androgens, and androgen receptors (ARs) compared with EDC effects on ERs. EDCs may act in a manner that primarily disrupts the balance between androgen and estrogenic signals, altering the endogenous ratio of testosterone, DHT, and 17β-estradiol synthesis (28). Urinary BPA concentration was inversely correlated to free androgen index in males (82) with evidence that BPA can potentially interfere with androgen production and function (83–85). EDCs did not affect functions of normal ARs, though it is possible that in certain disease states, such as prostate cancer, EDCs could influence patient therapy through mutant ARs (86, 87). Much work needs to be done to evaluate the differential effects that EDCs have on the various immune pathways important in disease management, tolerance, and autoantibody production in a sex-dependent context. It is possible that the actions exerted by various EDCs on androgens may reduce the immunoregulatory efficacy and tip the delicate balance toward promotion of autoimmunity.

**Figure 4:**
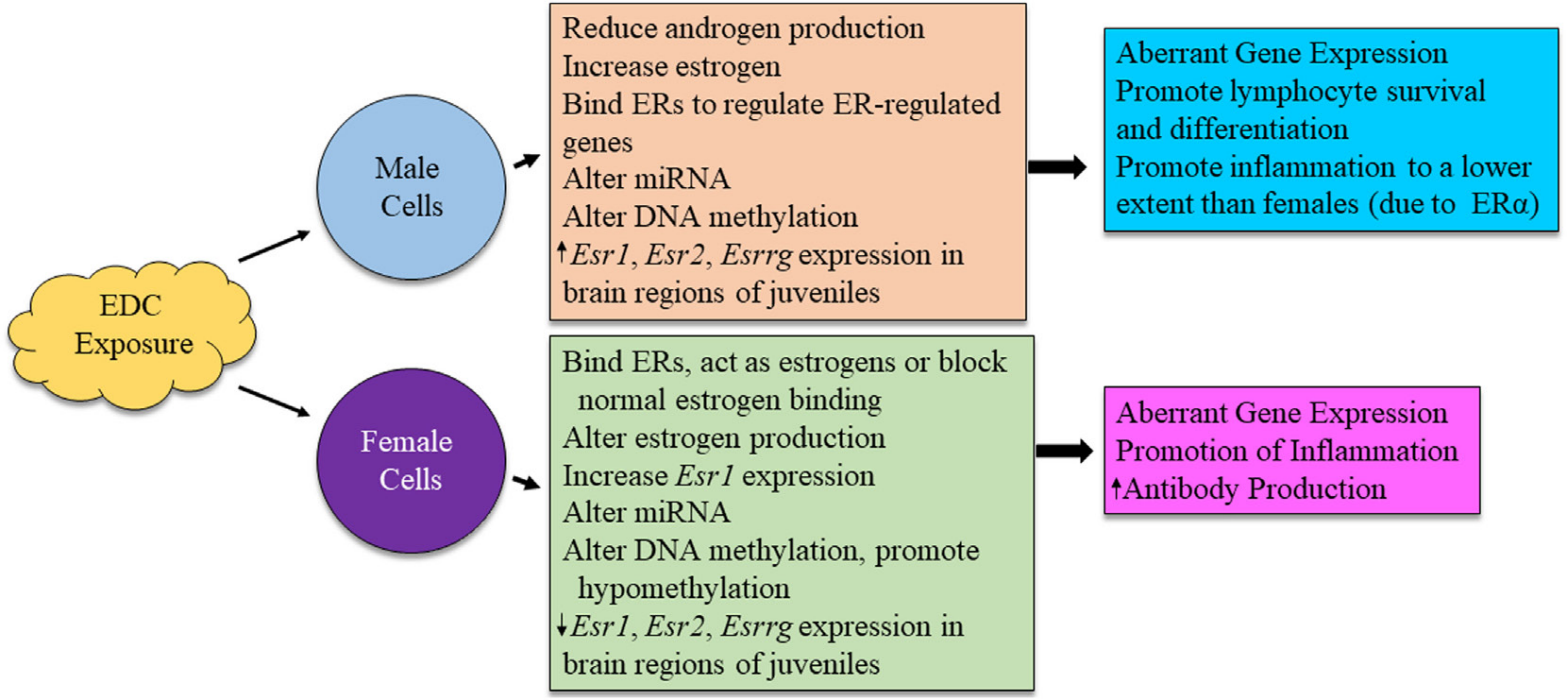
Possible mechanism for sex differences in environmental EDC exposure on immune function. The exact mechanism for immune system alterations due to EDC exposure in each sex is not yet well understood. Here, we propose possible mechanisms in which EDCs may exert sex-specific influence on immune cell functions.

Nevertheless, much work needs to be done to definitively understand the effects of endocrine disruptors on autoimmunity. Distinct sex-based responses to EDC exposure may contribute to dysregulation of the immune system to varying degrees in a disease-specific manner (Figure 4). Significant effort is still required to identify and molecularly characterize the impact of various environmental triggers, especially ubiquitous environmental contaminants, on the regulatory mechanisms of host immune systems.

### Androgens

The effects of DHT and testosterone in mammalian species have been shown to be primarily immunosuppressive (31, 88–91). ARs are expressed in lymphoid and non-lymphoid cells of the thymus and bone marrow. However, they have not been found in peripheral lymphocytes (92). This suggests that while androgens may not have a direct effect on lymphocyte function, they are important in developmental stages of T and B lymphocytes. Thymic epithelial cells and bone marrow stromal cells also act as mediators of androgen’s effects on immature lymphocytes (92). AR levels were not altered in the thymus following castration and were present on CD3^+^CD4^+^ and CD3^+^CD8^+^ thymic cells, with the highest level found on CD3^lo^CD8^+^ immature lymphocytes. ARs were also present in both cortical and medullary regions of the thymus following castration (92). The effects of castration extend to B cell development, leading to increased immature B cell populations in the bone marrow, as well as increased splenic B cells and enhanced antibody and autoantibody production in mice. Androgen replacement reversed the changes in the bone marrow, but did not affect splenic B cells (93).

**Figure 5:**
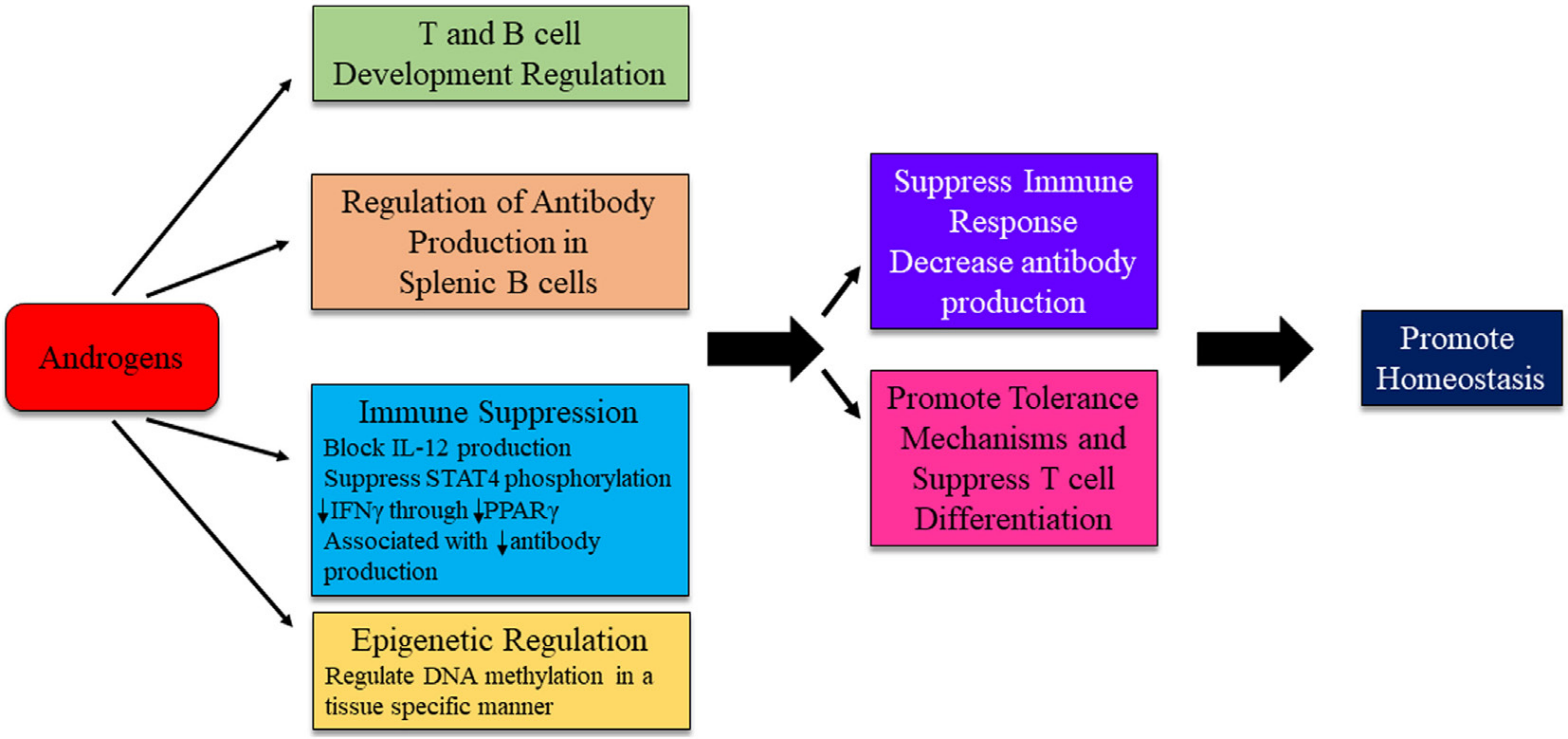
Possible mechanism for androgens to influence immunity and autoimmune disease development. The exact mechanism for androgenic influence on autoimmune disease development is likely disease and context dependent. Potential mechanisms of androgen regulation are depicted. Research is ongoing to identify distinct pathways in which androgens are able to exert effects on immune system regulation.

In general, there is good evidence that androgens downregulate immune system response in both normal and autoimmune individuals (Figure 5). Gonadectomized male mice, compared with intact females, had increased responses to, and reduced infection by, protozoans and fungi (31, 88–90). Androgens have been shown to suppress various autoimmune disorders including lupus and autoimmune thyroiditis (94, 95). Androgen deprivation led to increased T cell numbers (96). Inhibition of IL-12-induced STAT4 phosphorylation occurs through the AR binding to Ptpn1 conserved region, inhibiting IL-12 signaling in CD4^+^ T cells, and suppressing Th1 differentiation (97). Androgens reduce IFNγ production through decreased PPARγ (98). Suppressive effects are also exerted on B cell antibody production by androgens (99). In psoriatic arthritis patients, testosterone appears to exert a protective effect. Higher serum BAFF concentrations are associated with increased disease activity, while serum BAFF concentrations negatively correlate with circulating levels of testosterone (100). Therefore, it is possible that androgens can regulate the immune responses of a genetically autoimmune susceptible individual to favor the maintenance of homeostasis (Figure 5).

## Sex Difference in Stress Response and Autoimmunity

Associations have long been suspected between stressor events in a patient’s past and development of autoimmune diseases, such as SLE, MS, RA, and T1DM. Lack of evidence-based and prospective studies contribute to the skepticism that stressful life events are major etiopathological factors to consider in autoimmune disease development. Nevertheless, these events cannot be discounted, as stress responses can directly and indirectly influence immune responses. Sexual dimorphism exists in the hypothalamus–pituitary–adrenal (HPA) axis, a major component of the physiological stress response (101). Stress primarily acts upon the immune system through release of glucocorticoids, leading to alterations in cytokine production. In general, glucocorticoids inhibit the production of pro-inflammatory cytokines, such as IL-6, TNFα, and IFNα, whereas IL-4 and IL-10 are unaffected (102). Glucocorticoids are also able to inhibit the activation, proliferation, and differentiation of many cell types (103–106).

Fetal exposure to glucocorticoids can potentially impact a person’s HPA axis, either directly or indirectly; an effect to which female offspring are particularly vulnerable. Females exposed to a “prenatal stressor” had higher HPA reactivity than similarly exposed male offspring (107). Sex differences in cortisol response have been found in multiple life stages. Boys younger than 8 years of age had higher cortisol response than females of the same age. From 8 to 18 years of age, females have higher cortisol reactivity than males, an effect that is reversed in adulthood (107–112). Men have a more robust acute HPA response when compared with women, as determined by cortisol levels and sympathetic nervous system evaluation (113, 114). Men had higher glucocorticoid sensitivity and reduction in lipopolysaccharide (LPS)-stimulated cytokine production, whereas women had a decreased glucocorticoid sensitivity and increased LPS-stimulated cytokine production following a stress challenge (115). The type of stressor is also important when evaluating sexual dimorphism, as women had greater levels of cortisol in response to a social rejection challenge, while males had higher levels of cortisol in response to an achievement stimulus (116). Stress is able to alter plasma estradiol levels (117, 118) and estrogens have been shown to dampen the HPA and sympathetic nervous system response in certain studies (113, 119). However, other studies report a higher female HPA response independent of circulating gonadal hormone levels, suggesting either an innate difference in HPA mechanisms of action or an early developmental difference in response to sex hormone exposure (120).

A recent meta-analysis of 14 retrospective case–control studies supports major psychosocial stress as a risk factor for autoimmune disease development (121). This associated risk remained independent of the autoimmune disease reported. Appropriate controls in human studies exploring the role of stressors on autoimmune disease are difficult to determine, as the etiology of autoimmune diseases are still not well characterized. Most human retrospective studies rely on patient recall of stressful events that occur relatively close to disease diagnosis. It is possible that immune dysregulation and autoantibody production occur many years before the appearance of clinical signs. This would suggest that a stressor event that happens temporally close to the time of diagnosis would be a disease exacerbator, rather than an etiological factor. Consideration must also be given to the potential that the recrudescence of a latent virus or alteration in microbiota composition induced by a stressful event may be a driving factor in autoimmune disease development. Due to experimental limitations on human subjects, and the species differences in HPA axis response, these questions remain difficult to address, though the use of humanized rodent models may help to mitigate these limitations.

## Sex Differences in Epigenetic Regulation and Autoimmunity

Recent studies highlight the importance of epigenetic regulation in biological systems development and function. Abnormal epigenetic regulation, such as microRNA (miRNA) dysregulation and DNA hypomethylation, has been implicated in autoimmune diseases (122–128). Epigenetic mechanisms are important contributors to the balance between functional gene expression and regulation. These pathways, such as those that drive specific gene DNA methylation status, are dynamic processes and may potentially be altered in response to environmental cues and contaminants.

### Hormones Influence Epigenetic Regulation

Recent studies have suggested that sex hormones regulate immu-nity and autoimmunity through epigenetic mechanisms (Figures 3 and 5). miRNAs is a class of small non-coding RNAs that has emerged as a key epigenetic regulator of immune system functions in the last two decades (129). We have reported that estrogen regulated a set of miRNA in the splenic cells of normal B6 mice, of which, miR-146a and miR-223 were further validated to contribute to enhanced inflammation in splenocytes from estrogen-treated mice (130). Many estrogen-regulated miRNAs, such as miR-17-92, miR-125, miR-181a, miR-155, and miR-150, have been implicated in the regulation of B cell development and antibody production by targeting different genes such as Bim, C-Myc, Lin28, Pu.1, and AID (129, 131, 132). This suggests that estrogen may regulate B cell functions and antibody production *via* miRNA regulation. We recently reported that select lupus-associated miRNAs were differentially expressed in male and female NZB/W_F1_ mice and that estrogen promoted the expression of these lupus-associated miRNAs in orchidectomized male NZB/W_F1_ mice. Estrogen conferred a female expression pattern of miRNAs on the male NZB/W_F1_ mice, contributing to the female bias of lupus (36). Estrogen regulation of miRNA expression and the underlying mechanism has been further reviewed in more detail in our previous publication (5).

Increasing evidence indicates that sex influences the DNA methylome, which contributes to the sex differences in organ development, function, and susceptibility to specific diseases. Estrogen regulation of DNA methylation is suggested by the finding of the positive correlation between ER-positive status and promoter hypermethylation in breast tumors (133, 134). Estrogen has been reported to upregulate DNA methyltransferase (DNMT)3b expression in Ishikawa endometrial adenocarcinoma cells to facilitate malignant transformation of endometrial cancer cells (135). However, the inhibitory effect of estrogen on DNA methylation has also been observed in prostate cancer cell lines, which was mediated by the activation of ERβ, suggesting the importance of context on estrogen’s actions (136). There are limited data with regard to estrogen regulation of DNA methylation in immune cells. Autoimmune regulator (*AIRE*) is a negative regulator of autoimmunity, which is differentially expressed in the male and female thymus and contributes to the gender difference of autoimmune diseases (39). A recent study revealed that estrogen downregulated *AIRE* expression by inducing DNA methylation at the promoter, contributing to the female bias of autoimmune diseases (39). Nevertheless, the detailed mechanism of estrogen-mediated promotion of DNA methylation at the *AIRE* promoter remains to be clarified in future studies.

DNA methylation plays an essential role in regulation of sexual dimorphism of brain function during early development. It has been shown that females display higher DNMTs activity and hypermethylation in the highly sexually dimorphic preoptic area at postnatal day 1. Treatment with the testosterone metabolite estradiol significantly reduced global methylation at the preoptic area, leading to brain masculinization (137). Yolk testosterone was positively correlated with methylation levels of the ERα promoter in the diencephalon (138). The AR can both prevent DNA methylation through binding of the AR to the promoter of a gene of interest and promote DNA methylation through interaction of the AR with a suppressor, silencing expression of the gene of interest and eventual DNA methylation. AR function is associated with distinct DNA methylation patterns in genital tissues (139). Interestingly, the DNA methylation analysis of human blood revealed that there was a tendency of higher methylation levels in healthy males when compared with healthy females (140). Although the mechanism was unknown, we observed a reduction of global DNA methylation in splenocytes from estrogen-treated B6 mice when compared with placebo-controls. Given that DNA hypomethylation plays an important role in autoimmune diseases, such as lupus, it is significant to understand whether the gender difference in DNA methylation in immune cells contributes to the female bias of autoimmune disease directly and whether estrogen plays a role in the sexual dimorphism of DNA methylation in immune cells. It is noteworthy that sex hormones may regulate DNA methylation differentially in the context of different tissues, developmental stages, and pathological conditions. It should also be considered that the effect of estrogen on the global methylation level and the methylation of specific gene loci in defined subsets of cells of the immune system may be different.

### EDCs Influence Epigenetic Regulation

An individual’s ability to respond to an immunological stimulus can be modified generations before that individual is even conceived, primarily through the trans-generational effect of EDC exposure on epigenetic regulation of immune system development (141–143). After conception, maternal exposure to EDCs can also lead to alterations in the fetal epigenome, potentially leading to aberrant development of multiple body systems in the developing fetus (49). Following birth, that individual will continue to encounter EDCs through various sources and routes of exposure including, but not limited to, drinking water, cosmetics and personal hygiene products, handling of food containers and consumption of the stored contaminated food, medications, and pesticides (144–146). Exposure to these myriad EDCs can potentially alter an individual’s epigenome throughout all stages of life, influencing the body’s development and overall response to stimuli.

Endocrine disrupting chemicals have been shown to be involved in the three known forms of epigenetic regulation: miRNA production, DNA methylation, and histone modification. BPA is commonly used as a model EDC to investigate mechanisms by which estrogenic EDCs are able to modulate cellular functions. To date, most studies have focused on BPA’s ability to alter non-lymphoid tissue epigenetics. Dose and sex-specific changes were noted in ER gene expression, DNMT1 and DNMT3a expression, and DNA methylation status of the ERα gene *Esr1* in various areas of the brains of BALB/c mice exposed *in utero* to BPA. Male mice had increased *Esr1, Esr2, Esrrg, DNMT1*, and *DNMT3a* expression in the hypothalamus at low- and mid-range doses of BPA, but reduced expression at high doses, while the females showed the reverse effect. Female mice showed hypomethylation on multiple exons of the *Esr1* gene when exposed *in utero* (141). BPA exposure by pre-pubescent girls in Egypt led to evidence of hypomethylation of CpG-islands on the X-chromosome and reduced methylation levels in multiple genes associated with immune function (147). Overall, exposure to estrogenic EDCs, such as BPA, is associated with hypomethylation. CD4^+^ T cells have been shown to be hypomethylated in human SLE patients compared with healthy control (148). Therefore, it is possible that the reduced methylation associated with exposure to EDCs contributes to the hypomethylation of CD4^+^ T cells seen in SLE and systemic sclerosis patients, promoting aberrant gene expression in these cells, contributing to disease pathology.

Estrogenic environmental agent exposure can lead to aberrant miRNA expression profiles. BPA and DDT are able to alter the miRNA expression in a similar manner to estrogen. Increases have been seen in miR-21 and miR-146a. BPA was shown to decrease miR-134 (149–152). Lupus-prone MRL/*lpr* mice fed a chow-based diet containing phytoestrogens had increased expression of multiple miRNA and higher levels of global DNA methylation following LPS stimulation in splenic leukocytes along with increased DNMT1 expression (23). While the precise mechanism for this paradoxical finding is not yet known, it is possible that in select immune cell subsets, DNA methylation was reduced, or that the increased DNA methylation status suppressed immunoregulatory pathways, contributing to the enhanced disease phenotype seen in these mice. We are further investigating these findings. Long-term BPA exposure enhanced the expression and function of histone deacetylase 2 in adult mice, specifically in the hippocampus (153). Currently, there are no known effects of epigenetic regulation by BPA specifically on immune cell subsets. Further investigation is warranted into mechanisms by which estrogenic EDCs can alter the epigenome in immune cell subsets and promote tolerance dysregulation and antibody production.

## Autoimmunity and Microbial Agents

Observational relationships between infections and autoimmune diseases have long been recognized. Infections have been reported in a number of autoimmune diseases that either preceded overt expression of autoimmune disease or noted concurrently. Associations have been made between human CMV and Epstein–Barr virus (EBV) and autoantibody production in SLE patients, EBV and *Mycoplasma arthritidis* in RA patients, and multiple viruses, including hepatitis E virus, in type I diabetes mellitus (154–158). In most associations, antigenic mimicry is thought to be the mechanism that drives autoantibody production. It is evident that for the majority of vaccinations and viral pathogens, females mount a much stronger antibody response, suggesting that if subsets of female B cells were to break central and peripheral tolerance, that these abnormal B cells would drive higher autoantibody production than male autoreactive B cells. In the same manner as gene associations, it is very difficult to link a single infection, or multiple infections, with causation of autoimmune diseases. Alterations in commensal gut microbiota composition have been found in multiple mouse models of SLE as well as human SLE patients (22, 159). Breaks in the mucosal barrier during stressful events or during the female reproductive cycle may expose the immune system to both infectious and commensal microbes (160). As the role of infectious agents potentially contributing to autoimmunity has been well documented to date, this review will focus on recent evidence linking sex differences in response to microbial stimulation, commensal microbiota, and environmental factors that may influence autoantibody production in susceptible individuals.

### Sex Differences and Microbiota

The host microbiota, which has repeatedly been shown to influence immune phenotype, is dependent on multiple host factors, including age, diet, sex hormones, antibiotic usage, host genetics, obesity status, and various lifestyle choices. Early host–microbe interactions during childhood development can have long term and profound consequences on adult health through immune system “training” and induction of tolerance (161). Males and females have distinct microbial profiles, seen both in humans and mouse models of disease (162, 163). Gnotobiotic male and female C57BL/6 mice were administered the colonic contents of a human male. Upon analysis, the female mice had higher diversity as assessed by Shannon Diversity index, and a separate profile, whereas the male mice more closely resembled the donor profile. Forty-six distinct operational taxonomic units (OTUs) were different between the sexes, with 33 OTUs being overrepresented in the female fecal microbiota (162). Sex differences in microbial profiles were observed in multiple strains of mice. Gonadectomy with or without hormone replacement revealed further evidence of hormone effects on sex differences in mouse gut microbiota (164). In humans, the microbiota of males had reduced representation of *Bacteroides* at a BMI > 33, and the level of these microbes was reduced with increasing BMI. Post-menopausal females did not show an alteration in *Bacteroides* associated with BMI (165). An early critical window for microbial alteration of disease was shown for T1DM development in non-obese diabetic mice (NOD mice). Genetically similar mice housed in separate facilities eventually led to differing rates of T1DM development resulting in NOD^low^ and NOD^high^ communities. Co-housing or oral gavage of fecal contents from the NOD^high^ mice to NOD^low^ weanlings did not alter T1DM incidence. However, the offspring of the co-housed NOD^low^ mice did have increased T1DM incidence, suggesting that a window exists either *in utero* or before weaning where alterations of the microbiota result in disease development later in life (24). The importance of differing environmental, housing, and laboratory conditions on animal models of disease phenotypes is evident in this study. It is plausible that male and female epithelial and immune cells respond in a differential manner to microbial recognition during the formative periods in infancy and early childhood, and this differential response contributes to the distinct differences found in microbial composition in adulthood. It follows that if male and female cells inherently respond differently to microbial recognition during development, then exposure to EDCs during this important time period could drastically alter the microbial composition, thereby exerting long-term consequences on adult health from childhood exposures. Further investigation is vital to determine the role EDCs play in the development of microbial composition and resultant functional alterations in childhood and adulthood.

Communication between the host and commensal microbes occurs through multiple pathways, which are not yet well understood. Recognition of microbial-associated molecular patterns (MAMPs), production of soluble mediators, and interactions within the microbiota–gut–brain axis are thought to be the predominant methods of host–microbe communication. Resident immune cells at mucosal sites are able to recognize MAMPs and promote inflammation or induce regulation through Foxp3-positive T cell populations (166–168). Cytokines, metabolites, hormones, mucus, and anti-microbial peptides are all mediators produced by the host in response to the presence of microbes, while microbes release short chain fatty acids, polysaccharide A, formyl peptides, and d-glyceo-β-d-mannoheptose-1,7-bisphosphate (HBP), all of which can modulate the host response and microenvironment (169). It is currently understood that a two-way communication channel exists between the gut microbiota and the central nervous system. Microbes exert effects on the vagal afferents and enteric nervous system, and resultant stress responses act through the HPA axis to drive or dampen cortisol secretion. Cortisol acts both locally and systemically on immune cells, promoting the secretion of various cytokines and chemokines, which in turn alters gut permeability and intestinal barrier function. These actions alter the microbial composition within the gut (170, 171). Thus, complex mechanisms of communication between the host and microbiota, which is dependent on specific microbial composition, may promote either tolerance or inflammation, both locally and systemically in a context-dependent manner.

In the context of autoimmune disease, Markle et al. investigated the sex differences in microbiota in a mouse model of autoimmune T1DM. Transfer of the male microbiota to female mice led to systemic alterations in sex hormone levels and protection of female mice against development of T1DM (163). This protective effect conferred by the transfer of male microbiota to female mice was dependent on AR activity. Blockage of AR activity by the AR antagonist flutamide, attenuated the protection from insulitis, autoantibody production, metabolome changes, and the capacity of T cell transfer to confer autoimmune disease in NOD SCID mice. Bacteria are able to metabolize sex hormones, thereby regulating the balance between active and inactive hormones, and potentially modulating hormone function (172). Probiotics were shown to enhance antibody response to vaccines, potentially affecting the efficacy of oral vaccinations due to gut microbiota (173, 174). We and colleagues showed that in a mouse model of SLE, *Lactobacillus* spp. was inversely associated with disease severity. Supplementation with *Lactobacillus* spp. led to reduced IL-6 production, suppression of IgG2a production and glomerular deposition, and increased IL-10 in circulation along with increased Treg populations and decreased Th17 subsets. *Lactobacillus* was also associated with reduced renal pathology. These protective effects were seen in female and castrated male MRL/*lpr* mice, but not in intact MRL/*lpr* mice, suggesting that some microbial effects act in a sex-hormone dependent manner (22). Sex-based differences in microbiota and effects on disease development or progression in the context of sex-biased autoimmune diseases are summarized in Table 1.

**Table 1:** Sex-based differences in microbiota and autoimmune disease development.

Autoimmune disorder	Animal models	Microbiota phenotype	Outcome	Reference
Systemic lupus erythematosus (SLE)	MRL-lpr mice	Elevated *Lachnospiraceae* sp.	Associated with more severe glomerulonephritis	(23)

SLE	MRL-lpr mice	Administration of *Lactobacillus*	Reduced proteinuria and renal pathology, serum IgG2a, IL-10, and IgA in castrated males, not intact males	(22)
			Decreased circulating luteinizing hormone	

SLE	MRL-lpr mice	Female MRL-lpr have elevated *Lachnospiraceae* and reduced *Lactobacillus* compared to controls, males showed no difference from controls	Accelerated disease in females compared with males	(21)

Type-1 diabetes mellitus (T1DM)	NOD mice	Male microbiota and gavage of male microbiota to female mice	Reduce T1DM in association with functional androgen receptor	(163)
		Germ-free conditions	Attenuated sex differences in cumulative T1D (%)	

T1DM	NOD mice	Segmented filamentous bacteria monocolonized	Protected males but not females	(175)
		Specific pathogen free conditions and colonization with segmented filamentous bacteria	Increased blood testosterone levels in males compared to germ free	

Rheumatoid arthritis	HLA-DRB1 0401, 0402 mice	0401 *Clostridium*-like bacterium dominant, loss of age, and sex differences in microbiota	More susceptible to disease	(176)
		0402 *Porphyromonadaceae* and *Bifidobacteria* dominant, retain sex- and age-based microbiota differences	Resistant to disease	

### Sex Differences, Pathogen Sensing, and TLR7/8/9

Microbial signals are recognized through multiple pattern-recognition receptors, including TLRs, NOD-like receptors, C-type lectin receptors, and RIG-I-like receptors that are present in varying levels between cell subsets. Males and females tend to be exposed to the same pathogens and inflammatory triggers. However, the responses and outcomes to these exposures can be vastly different between sexes. One key sex difference in pathogen and inflammatory trigger sensing comes from the different levels of receptor expression. There are multiple immune-related genes, including TLR7, TLR8, FOXP3, CD40L, and CD13 that are present on the X-chromosome (177). It is possible that incomplete X chromosome inactivation can lead to increased numbers of FoxP3^+^ cells. Interestingly, while the number of FoxP3^+^ cells increased, the mean fluorescent intensity of FoxP3 was decreased and functional ability of these cells to regulate immune responses was suppressed (178). There is a potential for increased levels of TLR7 and 8 expression due to incomplete inactivation of the X-chromosome in females. The balance among TLR7, 8, and 9 has been shown to be vital to disease development in mouse models of SLE. TLR7 and 8 sense single-stranded RNA, and TLR9 binds to specific unmethylated CpG DNA motifs. Mice with increased levels of TLR7, or loss of TLR9, had enhanced autoantibody production and diseases severity, with TLR9 suppressing the autoantibody production induced by TLR7 (179). Male mice had higher levels of TLR4, circulating LPS-binding protein, and increased expression of CD14 on macrophages than female mice following LPS stimulation *in vivo*. These changes were seen at the protein level, while mRNA expression was unchanged between sexes (180, 181). Therefore, the potential differences in ability of male and female cells to recognize microbial or self stimuli may contribute to the observed differential responses.

Sex differences are also observed in how immune cells respond internally following ligand binding to receptors. Female Kuppfer cells produce IL-6 in a MyD88-dependent pathway, while male cells produce IL-6 in a MyD88-independent pathway (182). The ligation of CD200–CD200R is important in the suppression of TLR7 response to pathogens and control of IFNα production (183). Release of this inhibition through the knockout of the CD200 gene in mice enhanced sex differences in TLR7 and outcomes of viral infection. In HIV-1 infection, female plasmacytoid dendritic cells produced higher levels of IFNα after TLR7 stimulation than males and also had enhanced expression levels of all 13 IFNα subtypes and IFNβ following stimulation of TLR7 on peripheral blood mononuclear cells (PBMCs) (183). Caution must be taken when evaluating changes in expression levels and correlating that to immune responses, as PBMCs from human SLE patients showed increased expression of TLR9 mRNA and proteins compared with healthy controls. However, the function of TLR9 was impaired in SLE PBMCs leading to reduced IFNα following stimulation (184). Therefore, it is plausible that female cells may be more sensitive to PAMPs, and following receptor binding, internal signaling differences may enhance inflammatory responses compared with male cells.

## Conclusion and Future Directions

Biological differences exist in immunological responses to stimuli between males and females, and this likely contributes to the sex difference in the loss of immunological tolerance and production of autoantibodies. These differences manifest in a complex network of intrinsic differences in the ability of immune cells to recognize a stimulus, respond, and return to homeostasis. Female and male cells could have differential cell signaling and outcomes to environmental contaminants exposure, concurrent disease or pathogen exposure, commensal populations, age, sex hormone fluctuations, and other environmental influences. While this biological end point generally protects females from infectious diseases, it predisposes genetically susceptible individuals to chronic inflammatory conditions and development of autoimmunity compared with their male counterparts. Our understanding of environmental interactions with sex-specific characteristics remains incomplete, with evidence that sex-specific therapies or preventive measures may exist. Sex-based differences have been seen in response to treatment with methylprednisolone and rituximab (a monoclonal antibody against CD20) (185, 186). Sex disparities in clinical presentation, progression, and outcome of autoimmune diseases exist, suggesting that separation of sex-based groups in the evaluation of treatment strategies may help to appropriately tailor multiple treatment modalities in the future (187, 188). Environmental exposures may also influence an individual’s response to medication, highlighting the importance of considering a patient’s environment when determining the best possible treatment regimen. Further investigation into the precise mechanism of how environmental chemical exposure alters distinct ligand–receptor signaling cascades in specific immune cell subsets is vital for better understanding the extent to which these ubiquitous chemicals can modify human and animal physiology. The exploration into associations between commensal microbial dysregulation and host disease susceptibility, or severity, must also take into account, to the best extent possible, the environment to which that particular individual has been exposed. Microbial metabolism of EDCs may exert protection or promote exacerbation of certain disease processes depending on resultant metabolites and bioavailability.

**Figure 6:**
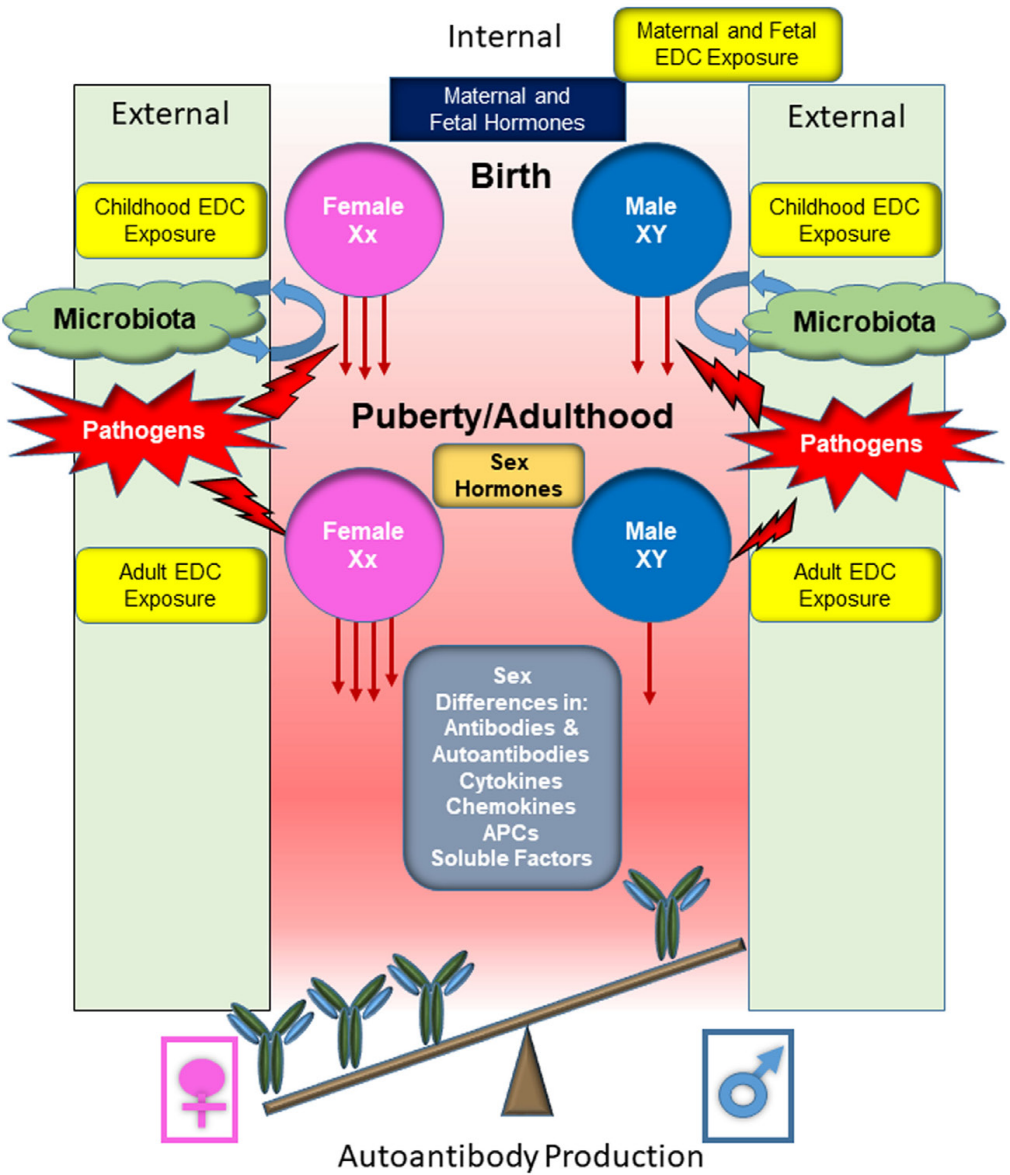
Overview of the responses by male and female B cells to external stimuli and subsequent production of autoantibodies. Before conception, the maternal systems are augmented in various ways, including exposure to endocrine disrupting chemicals (EDCs). Following conception, the fetus is subjected to EDC exposure through continued maternal exposure. Fetal and maternal hormones contribute to immune system development. Following parturition, an infant becomes exposed to microbial organisms and the establishment of commensal microbial population begins, being influenced by both endogenous and exogenous factors before stabilization. The microbiota composition will influence the developing immune system and promote recognition of pathogens and support the development of tolerance mechanisms. Juveniles continue to be exposed to EDCs and pathogenic microbes will be encountered, further stimulating the immune system. At puberty, cells become exposed to higher levels of female or male sex hormones, altering immune cell function and signaling pathways. During adulthood, immune cells continue to be exposed to EDCs and microbial stimuli, both commensal and pathogenic. Sex differential levels of cytokines, chemokines, hormones, and other soluble factors make up the microenvironment that the immune cells are exposed to, further promoting or inhibiting immune cell activation and response. The female cells, which may be primed due to sex differences and environmental contributing factors, generally respond more robustly to the same immunological stimulus compared with male cells. This more robust response likely contributes to the female biased dominance in many autoimmune diseases.

Given that multi-factors are likely required for the induction of autoimmune diseases, a different approach is needed to understand sex differences in susceptibility to these chronic conditions. Biologically, male and female cells of the immune system are differentially exposed to sex hormones and sex hormone-regulated proteins (other unidentified internal regulators) and manifest inherent genetic differences in sex chromosomes. Thus, the cells of the innate and adaptive immune system could be molecularly primed differently between sexes. It is therefore conceivable that male and female cells will differently perceive the binding of their receptor to the same ligand. In females, exposure to these triggers may have adverse effects. Following external triggers, the female immune system may be more prone to dysregulation (e.g., break in immune tolerance) and have augmented induction of autoantibodies and cytokines/chemokines that have been associated with autoimmune diseases. We further postulate that in genetically susceptible individuals, sex differences, both intrinsic and in response to environmental contaminants, such as estrogenic EDCs, contribute to female bias of immune tolerance dysregulation and drive autoantibody production and subsequent pathology (Figure 6). Understanding the complex interactions among differences in sex hormones, a wide range of chemicals, genetic variations, infections, and environmental triggers and deriving conclusions about impacts on pathogenesis will likely require the utilization of complex computational models. With the progression of individualized medicine, these environmental exposures will likely prove unique to various lifestyles and geographical locations.

## Author Contributions

All authors listed have made a substantial, direct, and intellectual contribution to the work and approved it for publication.

## Conflict of Interest Statement

The authors declare that the research was conducted in the absence of any commercial or financial relationships that could be construed as a potential conflict of interest.

## Abbreviations

AID, activation-induced cytidine deaminase; AIRE, autoimmune regulator; AR, androgen receptor; BAFF, B-cell-activating factor; BPA, bisphenol-A; CLRs, C-type lectin receptors; CMV, cytomegalovirus; DHT, dihydrotestosterone; DNMT, DNA methyltransferase; EBV, Epstein–Barr virus; EDC, endocrine disrupting chemicals; ER, estrogen receptor; GPR, G protein-coupled estrogen receptor; HDAC, histone deacetylase; HEV-hepatitis E virus; IFN, interferon; LPS, lipopolysaccharide; MAMPs, microbial-associated molecular patterns; miRNA, microRNA; MRL/lpr, MRL/MpJ-Faslpr/J; MS, multiple sclerosis; NLR, NOD-like receptor; NZB/W_F1_, New Zealand Black/White F1 progeny; NOD mice, non-obese diabetic mice; PBMCs, peripheral blood mononuclear cells; pDCs, plasmacytoid dendritic cells; PPAR, peroxisome proliferator-activated receptor; RLRs, RIG-I-like receptors; SCFA, short chain fatty acid; SLE, systemic lupus erythematosus; T1DM, type-1 diabetes mellitus; TLR, toll-like receptor.
